# Shared and distinct molecular signatures of astrocyte subtypes across brain regions and neurodegenerative diseases

**DOI:** 10.1002/alz.093351

**Published:** 2025-01-03

**Authors:** Ibrahim Olabayode Saliu, Guoyan Zhao

**Affiliations:** ^1^ Washington University School of Medicine, St. Louis, MO USA; ^2^ Washington University School of Medicine, St Louis, MO USA

## Abstract

**Background:**

Astrocytes are specialized glial cells that play crucial roles in the brain by providing metabolic and trophic support for the neurons. They become reactive (activated) and heterogeneously respond to neuropathology such as injuries and neurodegenerative diseases (NDs). High‐throughput single‐nucleus (snRNA‐seq) RNA sequencing has enabled a profound understanding of cell type heterogeneity. However, the number of astrocyte subpopulations in different regions of the brain, and the similarities and differences among these subpopulations in healthy and disease conditions are not clear.

**Method:**

We performed uniform data analysis using the same algorithm and parameters on astrocytes from postmortem human brain single‐nucleus RNA‐sequencing (snRNA‐seq) data obtained from eight datasets with seven different brain regions of normal control subjects and subjects with different NDs including Alzheimer’s disease (AD), dementia with Lewy body disease (DLBD), Parkinson’s disease (PD), Parkinson’s disease with dementia (PDD), Huntingtin disease (HD), or Nasu‐Hakola disease (NHD). We identified astrocyte subpopulations and systematically compared astrocyte subpopulations and defined their common marker gene set across seven brain regions in six disease conditions. We conducted functional enrichment pathway analysis on the marker genes to understand the putative contribution of these subpopulations to NDs including across different brain regions.

**Result:**

We identified 3‐6 distinct astrocyte subpopulations by examining the expression of conserved marker genes across all the different datasets. Two subpopulations were shared across all the NDs and brain regions, representing homeostatic (Ast‐0) and activated transcriptional states (Ast‐1 and Ast‐2), while the other subpopulations are unique to different datasets. Functional enrichment analysis revealed distinct and shared pathways among astrocyte subpopulations across all brain regions.

**Conclusion:**

We identified astrocyte subpopulations shared across all the brain regions and disease conditions with shared molecular marker genes but also regional‐ or disease‐specific gene expression features. We also observed disease‐ or brain region‐specific astrocyte subpopulations. These results suggest that regional differences in astrocyte subpopulations or gene expression could underly regional differences in brain susceptibility to different neurodegenerative diseases.

